# Low-molecular-weight paramagnetic ^19^F contrast agents for fluorine magnetic resonance imaging

**DOI:** 10.1007/s10334-018-0721-9

**Published:** 2018-11-29

**Authors:** Vít Herynek, Marie Martinisková, Yulia Bobrova, Andrea Gálisová, Jan Kotek, Petr Hermann, Filip Koucký, Daniel Jirák, Milan Hájek

**Affiliations:** 1 Center for Advanced Preclinical Imaging, First Faculty of Medicine, Charles University, Salmovská 3, Prague, Czech Republic; 2 MR-Unit, Department of Diagnostic and Interventional Radiology, Institute for Clinical and Experimental Medicine, Vídeňská 1958/9, Prague, Czech Republic; 3 Department of Inorganic Chemistry, Faculty of Science, Charles University, Hlavova 2030, 12843 Prague 2, Czech Republic; 4 Department of Low Temperature Physics, Faculty of Mathematics and Physics, Charles University, V Holešovičkách 2, Prague, Czech Republic

**Keywords:** Fluorine-19 magnetic resonance imaging, Molecular probes, Lanthanide series elements, Macrocyclic ligand complexes, Phosphinic acid complexes, Relaxation times

## Abstract

**Objective:**

^19^F MRI requires biocompatible and non-toxic soluble contrast agents with high fluorine content and with suitable ^19^F relaxation times. Probes based on a DOTP chelate with 12 magnetically equivalent fluorine atoms (DOTP-tfe) and a lanthanide(III) ion shortening the relaxation times were prepared and tested.

**Methods:**

Complexes of DOTP-tfe with trivalent paramagnetic Ce, Dy, Ho, Tm, and Yb ions were synthetized and characterized. ^19^F relaxation times were determined and compared to those of the La complex and of the empty ligand. In vitro and in vivo ^19^F MRI was performed at 4.7 T.

**Results:**

^19^F relaxation times strongly depended on the chelated lanthanide(III) ion. *T*_1_ ranged from 6.5 to 287 ms, *T*_2_ from 3.9 to 124.4 ms, and *T*_2_* from 1.1 to 3.1 ms. All complexes in combination with optimized sequences provided sufficient signal in vitro under conditions mimicking experiments in vivo (concentrations 1.25 mM, 15-min scanning time). As a proof of concept, two contrast agents were injected into the rat muscle; ^19^F MRI in vivo confirmed the in vivo applicability of the probe.

**Conclusion:**

DOTP-based ^19^F probes showed suitable properties for in vitro and in vivo visualization and biological applications. The lanthanide(III) ions enabled us to shorten the relaxation times and to trim the probes according to the actual needs. Similar to the clinically approved Gd^3+^ chelates, this customized probe design ensures consistent biochemical properties and similar safety profiles.

## Introduction

Magnetic resonance imaging (MRI) is a technique commonly used for clinical and preclinical imaging. Basically, magnetic resonance may detect all the isotopes with an odd number of protons and/or neutrons. Most often, a proton (^1^H) signal is detected, because all the tissues contain a substantial amount of water. However, other nuclei present in the living systems, such as ^31^P, ^13^C, and ^23^Na, among others, are also interesting targets for imaging or spectroscopic techniques [1]. Although amount of fluorine (^19^F) in living organisms is negligible, ^19^F MRI is a potentially interesting tool for preclinical or even clinical imaging [2, 3], as long as a suitable fluorinated tracer is used as a contrast agent. Natural fluorine is monoisotopic, and ^19^F nucleus has a resonance frequency close to the proton frequency. Therefore, standard commercial scanners can be used for fluorine detection after introducing only minor hardware and software changes. Moreover, because the concentration of MR-detectable fluorine in living organisms is negligible, fluorine-based images have no background. Thus, in combination with standard ^1^H MRI, this approach provides the so-called “hot-spot imaging”, which is particularly applicable to cell tracking [4, 5].

Despite the potential of ^19^F MRI, this method has some caveats. Although the signal intensity of fluorine is similar to that of hydrogen, its concentration in the synthetized probes is significantly lower. While the concentration of water ^1^H in the human body is approximately 70 M, concentration of any externally added ^19^F tracer will be in the order of mM only. Hence, to generate a detectable ^19^F MRI signal, several probe designs and technical approaches have been combined, albeit with some drawbacks specific to each approach [6]. Probe design has been improved to increase the signal strength by binding more fluorine atoms to a probe molecule, such as in perfluorocarbons [7] (for example, perfluoro-15-crown-5-ether with 20 magnetically equivalent fluorine ions has been often used [8]) or in superfluorinated probes [9]. However, this approach may decrease the solubility of the contrast agent because these liquids are immiscible with water. Alternatively, to overcome these solubility problems, liposomal [10] or polymer [11] nanoparticles containing fluorinated molecules have also been tested in various applications, particularly in cell tracking [12, 13]. These nanoparticles are usually cleared through the reticulo-endothelial system, and macrophage cytokines are released upon high repetitive dosage [14]. In contrast, low-molecular-weight fluorinated molecules can be used, e.g., as blood pool agents. Herein, the chemical structure of the probes developed in this study is similar to that of the commercially available Gd^3+^ chelates used for ^1^H MRI [15]. Therefore, our probes should have similar biochemical properties and safe renal clearance.

Technical approaches have also been used to improve sensitivity. Increasing voxel size decreases spatial resolution. Increasing the number of acquisitions substantially prolongs the overall measurement time. Generally, the long relaxation times of ^19^F nuclei require long repetition times, thus leading to extremely long acquisition times. However, relaxation times are substantially shortened when introducing a paramagnetic ion near the measured nucleus [16]. This approach may considerably shorten the relaxation times down to the millisecond range, thereby enabling the use of fast acquisition sequences and substantially enhancing the sensitivity [6, 17, 18]. Hence, transition metal ion complexes have been used as molecular ^19^F MRI contrast agents [19–23], although complexes of lanthanide(III) ions have been suggested more frequently [18, 23, 24].

Thus, in this study, we designed fluorinated probes based on chelates of lanthanide(III) ions with a new macrocyclic ligand, DOTP-tfe (DOTP-tfe = 1,4,7,10-tetraazacyclododecane-1,4,7,10-tetrakis[methylene(2,2,2-trifluoroethyl)phosphinic acid]), with 12 magnetically equivalent fluorine atoms. The complexes were synthetized, characterized, and tested both in vitro and in vivo with optimized imaging sequence timing.

## Materials and methods

### Ligand and complex syntheses

**Fig. 1:**
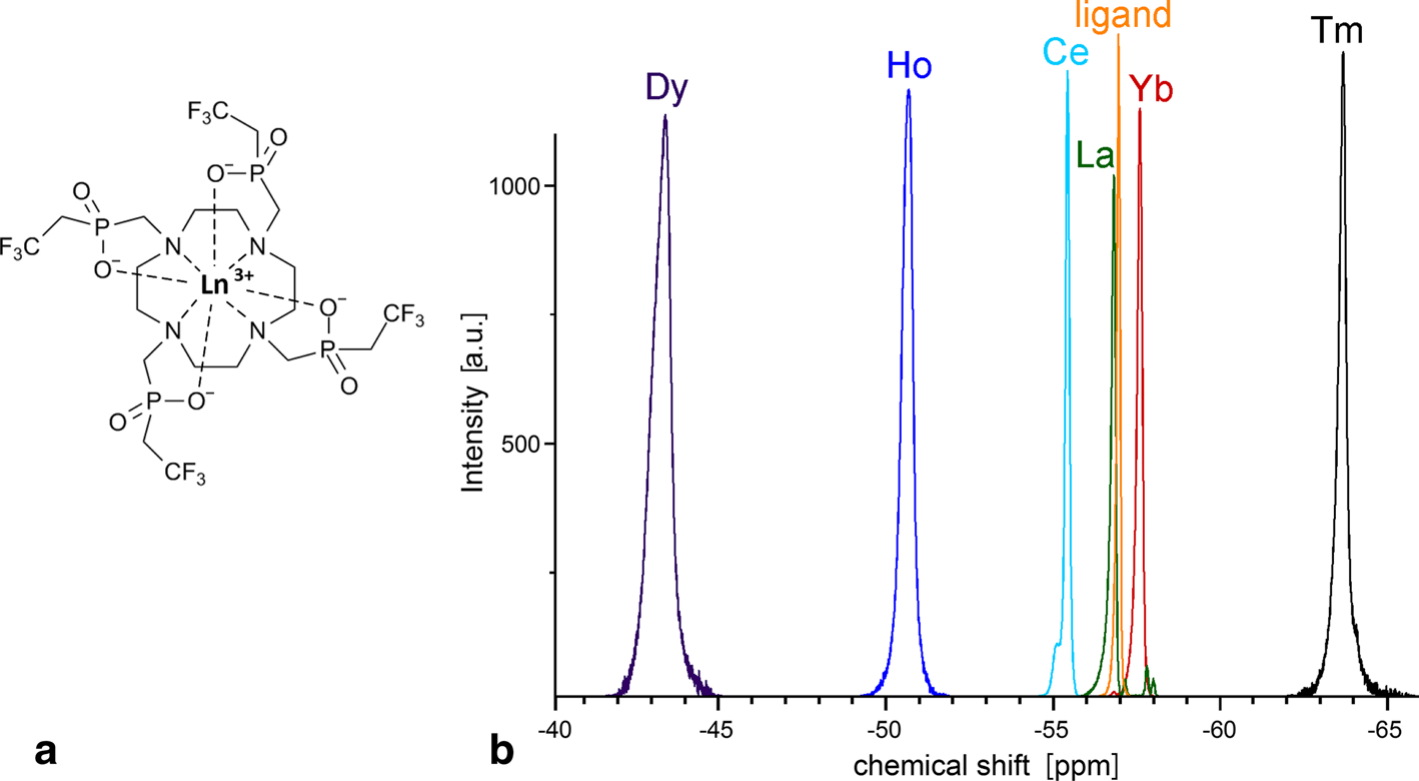
Structure of the Ln^3+^–DOTP-tfe complexes (**a**). Ln^3+^ = La, Ce, Dy, Ho, Tm, and Yb. Visualization of their NMR spectra showing chemical shift and line broadening caused by the complexed lanthanide(III) ions (**b**)

The ligand, DOTP-tfe, was synthesized by Mannich-like reaction of cyclen (1,4,7,10-tetraazacyclododecane) (2,2,2-trifluoroethyl)phoshinic acid (8 equiv.) and formaldehyde (10 equiv.) in aqueous HCl (pH 1) at 60 °C for 3 days, and the chelator was purified on ion-exchange resins [25]. The trivalent lanthanum (La), cerium (Ce), dysprosium (Dy), holmium (Ho), thulium (Tm), or ytterbium (Yb) ion complexes (Fig. 1) were prepared by mixing the aqueous solutions of the Ln(III) chlorides (1.5 equiv.) and of the ligand. The solution pH was adjusted to 5–6 with diluted aqueous NaOH and the solution was stirred at 50 °C for 3 days. The solution pH was maintained at ~ 6 by periodically adding NaOH solution until no pH drop was observed. The solution was vacuum dried, the solid residue was redissolved in water, and the solution was loaded onto Dowex 50 resin (1 × 3 cm column, H^+^-form). The pure complex was eluted by water. The solution was vacuum dried, and the solid residue was redissolved in water to prepare a 20 mM stock solution of the complex, which was then appropriately diluted for the following relaxation and MRI measurements. The solution was filtered through a syringe filter (0.22 µm) before use.

### NMR spectroscopy

High-resolution ^19^F NMR spectra of the DOTP-tfe ligand and its complexes with trivalent La, Ce, Dy, Ho, Tm, and Yb were acquired on Varian VNMRS300 NMR spectrometer with a fluorine frequency of 282.22 MHz. The paramagnetic complexes were measured with an acquisition time at = 600 ms, recycle delay d1 = 200 ms, pulse width pw =7 µs, and number of transients nt =128. The diamagnetic complex (La) and empty ligand were measured with at =3.82 s, d1 = 5.00 s, pw =10 µs, and nt =16. Chemical shift of the standard in an insert capillary (1.2 M CF_3_CO_2_H) was set to − 75.6 ppm.

The stability of the Dy^3+^ complex was measured in standard phosphate buffer saline (PBS: 4.0 g NaCl, 0.10 g KCl, 0.72 g Na_2_HPO_4_, 0.12 g KH_2_PO_4_ in 500 mL of water) and in rat blood serum by ^19^F NMR. The samples were incubated at 37 °C for 10 days. ^19^F NMR spectra were measured at the beginning of incubation, after 2 h, then once per day. The measurement parameters used were the same as those described above.

### Relaxometry

Relaxation times of the complexes were measured at 4.7 T Bruker imager (Bruker BioSpin, Germany) with a tunable 4-cm ^1^H/^19^F homemade single-loop surface coil. The *T*_1_ relaxation time was measured using a saturation recovery sequence with a recovery time TR in the range 32–4000 ms for the empty ligand and for the La complex, 15.5–1600 ms for the Ce and Yb complexes, 0.39–50 ms for the Tm and Dy complexes, and 0.39–100 ms for the Ho complex. Relaxation times *T*_2_ of the empty ligand, La, Yb, and Ce complexes were measured using a Carr–Purcell–Meiboom–Gill (CPMG) sequence with echo spacing TE = 7.214 ms and repetition time TR = 4000 ms (empty ligand and La complex) and TE = 6.78 ms and repetition time TR = 1000 ms (Yb, Ce complex). Short *T*_2_ relaxation times of the Tm, Dy, and Ho complexes were measured using a series of single-echo sequences with TE in the range 2.22–20 ms (Tm and Dy) or 2.5–30 ms (Ho). The data were fitted using in-house software ViDi (Matlab, MathWorks, Natick, MA, USA) and GNUPLOT (https://www.gnuplot.info). The *T*_2_* was estimated from the spectral linewidth. Measurements were repeated three times. The errors of the *T*_1_ and *T*_2_ values represent the standard error of the fit. For *T*_2_* data, average values of three measurements are given together with standard deviation.

Concentrations of the samples for in vitro measurements were at least in mM range; however, fluorine relaxation times do not depend on concentration.

### In vitro imaging

Gradient and turbo spin echo sequences were optimized to match the actual relaxation times of the complexes. For the Yb–DOTP-tfe and Ce–DOTP-tfe complexes, the turbo spin echo sequence with TE = 7.24 ms, turbo factor 20 (Ce) or 16 (Yb), repetition time TR =500 ms (Ce) or 200 ms (Yb), number of acquisitions NA =1000 (Ce) or 2000 (Yb), and matrix 32 × 32 and voxel size 1.5 × 1.5 × 2 mm^3^ were used. The gradient echo sequence was used for the Dy, Ho, and Tm complexes with following parameters: TE =3.19 ms, TR =15 ms, NA =2000 and same geometry as above. Both sequences took approximately 15–19 min which would be also reasonable for in vivo experiments. Complexes were measured at the tracer concentrations, from 0.625 mM (i.e., 3.9 mM of ^19^F) to 5 mM (i.e., 60 mM of ^19^F) and signal-to-noise ratio (*S*/*N*) was evaluated.

### In vivo experiments

To prove the principle, the contrast agents with a longer relaxation time, Yb–DOTP-tfe and Ce–DOTP-tfe were injected into a muscle in the hind leg of rats (0.5 mL; 2.7 mM) and imaged using the 4.7 T MR imager used in the in vitro study. Based on the relaxation times, the turbo spin echo sequence was optimized as follows: TE =7 ms, TR =500 ms, turbo factor 16, matrix 32 × 32, voxel size 1 × 1 × 10 mm^3^, NA =1024, and scan time 17 min. The ^19^F images were interpolated to 256 × 256 matrix and merged with standard *T*_2_-weighted anatomical ^1^H images.

The Dy, Ho, and Tm complexes requiring fast sequence timing could not be tested in vivo due to the hardware limitations of the used scanner. With these metal ions, loss of signal during the delay between excitation and data acquisition (minimal available TE = 3.19 ms) at in vivo conditions (lower probe volume and concentration, and possible dispersion of the probe in the tissue) did not enable to properly set the transmission pulse, which is crucial with respect to strong *B*_1_ inhomogeneity caused by the surface coil.

The rats were anesthetized by intramuscularly administered anesthetics (ketamine 36 mg/kg and dexmedetomidine 0.08 mg/kg) in both procedures (contrast agent application and MR scanning) to avoid the inhalation of a fluorine-based anesthetics.

## Results

Paramagnetic lanthanide(III) ions bound in complexes strongly influence both ^19^F NMR chemical shift (see Fig. 1b) and the ^19^F relaxation times, as summarized in Table 1. Ce and Yb moderately decreased both *T*_1_ and *T*_2_ relaxation times of the fluorine nuclei in the complexes, whereas Dy, Ho, and Tm decreased the relaxation times down to the low millisecond range, thus enabling to profit from the use of ultrafast acquisition sequences. Interestingly, diamagnetic La^3+^ has small effect on *T*_1_ relaxation time, but a stronger effect on *T*_2_ relaxation time (stronger than that caused by paramagnetic Ce^3+^ or Yb^3+^).

**Table 1:** The ^19^F relaxation times of the empty ligand and trivalent cerium (Ce), dysprosium (Dy), holmium (Ho), thulium (Tm), and ytterbium (Yb) complexes with DOTP-tfe

Complex	*T*_1_ (ms)	*T*_2_ (ms)	*T*_2_* (ms)
Empty ligand DOTP-tfe	1103 ± 49	363 ± 23	5.10 ± 0.20
La–DOTP-tfe	835 ± 26	53.8 ± 2.6	3.72 ± 0.13
Ce–DOTP-tfe	287 ± 46	124.4 ± 0.9	3.13 ± 0.16
Dy–DOTP-tfe	6.9 ± 0.3	3.9 ± 0.6	1.14 ± 0.06
Ho–DOTP-tfe	9.8 ± 0.3	8.2 ± 0.3	1.10 ± 0.06
Tm–DOTP-tfe	6.5 ± 0.4	4.9 ± 1.0	1.36 ± 0.07
Yb–DOTP-tfe	76 ± 9	72.1 ± 1.0	1.44 ± 0.07

**Fig. 2:**
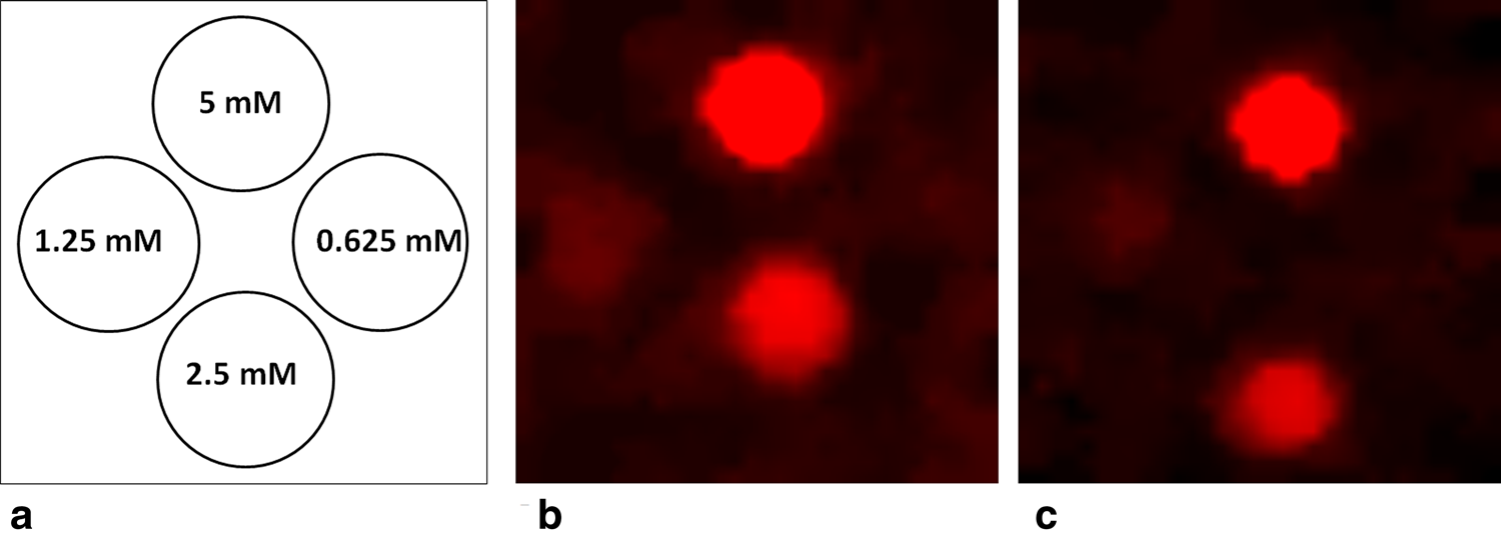
^19^F MRI of phantoms containing different concentrations of two selected complexes. **a** Arrangement of the test tubes with various concentrations of the complexes. **b** Ce–DOTP-tfe complex measured by a turbo spin echo sequence. **c** Dy–DOTP-tfe complex measured by a gradient echo sequence. Concentrations of the complexes down to 1.25 mM (corresponding to 15 mM of ^19^F) were detectable within a 15–20 min acquisition time in both cases

According to the relaxation times, turbo spin echo (for empty ligand, diamagnetic La–DOTP-tfe, and paramagnetic Yb–DOTP-tfe and Ce–DOTP-tfe complexes) or gradient echo (for paramagnetic Dy–DOTP-tfe, Ho–DOTP-tfe, and Tm–DOTP-tfe complexes) imaging sequences were optimized as described in the “Materials and methods” section. Figure 2 shows in vitro images of phantoms containing different concentrations of the Ce–DOTP-tfe complex measured by turbo spin echo sequence (Fig. 2b) and phantoms with the Dy–DOTP-tfe complex measured by gradient echo sequence (Fig. 2c). A signal was reliably detected at the concentrations of 1.25 mM (i.e. 15 mM ^19^F) and higher under conditions mimicking the experiments in vivo (a number of acquisitions were set to reach 15–19 min scan time). Signal-to-noise ratios of the phantoms containing the complexes at four different concentrations are listed in Table 2. Sequence optimization led to a similar signal-to-noise ratios in all complexes tested in this study.

**Table 2:** Signal-to-noise ratios of phantoms containing trivalent cerium (Ce), dysprosium (Dy), holmium (Ho), thulium (Tm), ytterbium (Yb), and lanthanum (La) complexes of DOTP-tfe in vitro

Probe	Probe concentration			
	0.625 mM	1.25 mM	2.5 mM	5 mM
Empty ligand	2.2 ± 0.1	2.6 ± 0.2	4.9 ± 0.3	9.4 ± 0.6
La–DOTP-tfe	2.1 ± 0.1	3.3 ± 0.2	5.2 ± 0.4	11.9 ± 0.8
Ce–DOTP-tfe	2.1 ± 0.5	3.2 ± 0.8	7.1 ± 1.8	13.6 ± 3.4
Dy–DOTP-tfe	1.5 ± 0.3	3.8 ± 0.7	9.3 ± 1.8	14.7 ± 2.9
Ho–DOTP-tfe	2.1 ± 0.2	3.5 ± 0.3	7.4 ± 0.6	14.3 ± 1.1
Tm–DOTP-tfe	2.4 ± 0.3	3.6 ± 0.4	9.5 ± 1.1	16.5 ± 2.0
Yb–DOTP-tfe	2.1 ± 0.6	4.0 ± 1.1	7.3 ± 2.0	15.3 ± 4.2

The empty ligand is added for comparison. The MRI data were acquired with optimized imaging sequences with 15–20 min scanning time

The complexes with longer relaxation times (La, Yb, Ce) as well as the empty ligand measured using a gradient echo sequence provided a signal at the detection threshold (*S*/*N* ~ 1.5) at the highest concentration (5 mM, i.e., 60 mM ^19^F) even after optimization (decreasing of a flip angle down to 30° together with decreasing TR to 120 ms and 256 acquisitions). Similarly, short relaxation times of the paramagnetic complexes (Dy, Ho, Tm) did not allow to use a turbo spin echo sequence. Therefore, direct comparison of the complexes was not possible.

The stability of a selected complex (Dy–DOTP-tfe) was tested by dilution in a phosphate buffer saline and rat blood serum. ^19^F NMR spectroscopy revealed no free ligand signal during the 10-day incubation at 37 °C, thus confirming that the complex is stable and that no lanthanide(III) ions are released from the complexes.

**Fig. 3:**
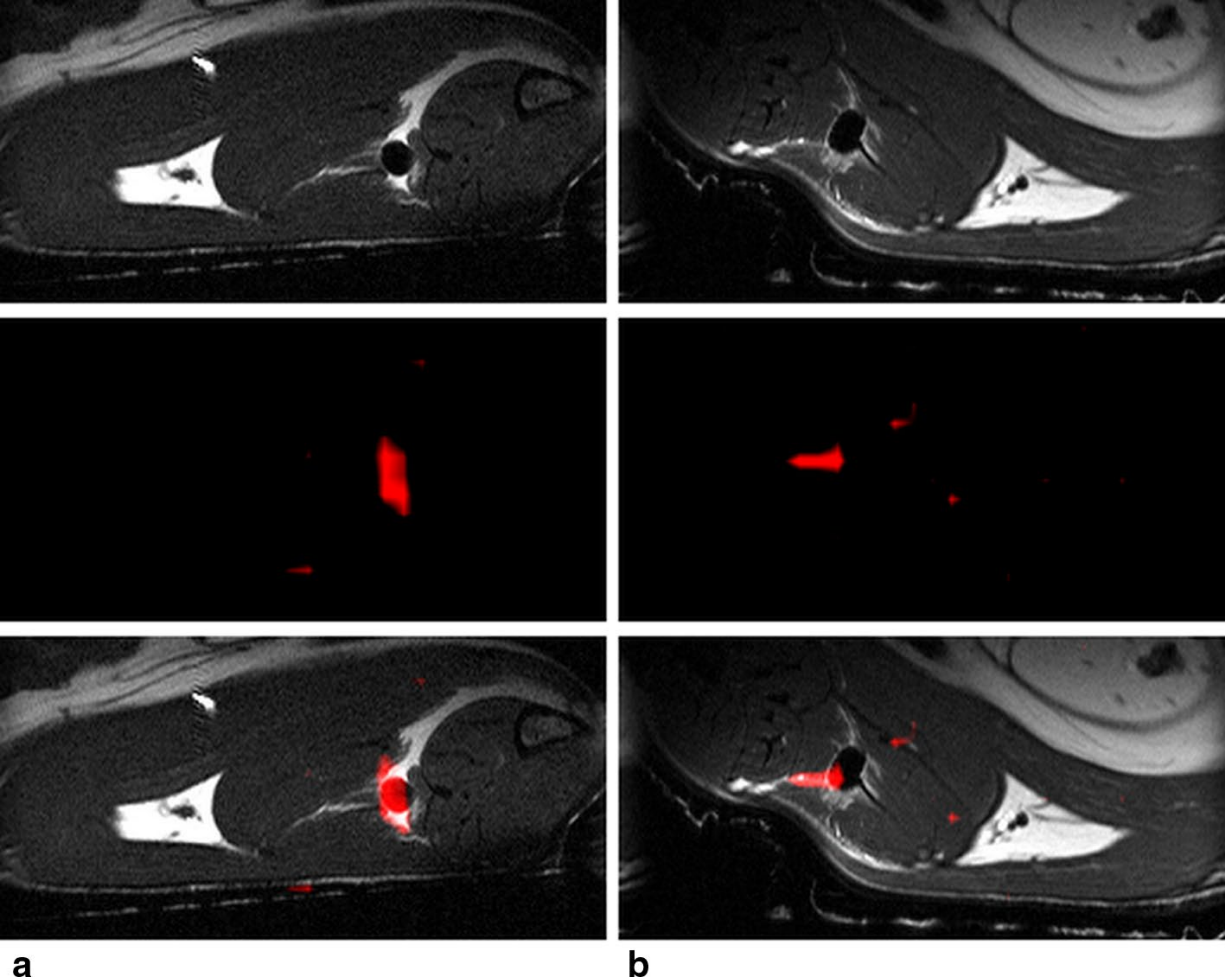
MR images of two different probes injected into the muscle of a healthy rat. **a** Yb–DOTP-tfe complex (0.5 mL, concentration 2.7 mM). **b** Ce–DOTP-tfe complex (0.5 mL, concentration 2.7 mM). First row—anatomical T_2_-weighted ^1^H MRI images (gray scale); second row—^19^F MRI (red color) extrapolated to the same matrix; third row—overlay of the ^1^H and ^19^F MR images. Note the hypointense signal on the ^1^H MRI images caused by the probe. Small displacement of the ^19^F signal in the case of Ce–DOTP-tfe in **b** may be caused by the extrapolation of the low image matrix of ^19^F MRI or by probe diffusion during the scanning

The in vivo application of the contrast agents into the muscle of the rat confirmed visibility of the Ce–DOTP-tfe and Yb–DOTP-tfe probes at the application site at concentrations down to 2.7 mM (see Fig. 3).

## Discussion

Although the sensitivity of ^19^F in magnetic resonance is similar to that of hydrogen, the signal level is the main problem of ^19^F MR imaging. Any probe concentration may safely reach even the millimolar range, which is still incomparably lower than the concentration of water hydrogen atoms in tissues. Therefore, molecules with the high number of fluorine atoms per molecule of ^19^F MRI contrast agent are desirable targets [6]. Symmetrical perfluorinated organic molecules could be such targets, but these molecules are, generally, insoluble in water and can be used only in nanoparticles, such as micelles. Here, we have overcome this limitation by designing a probe with 12 magnetically equivalent fluorine nuclei. Although replacing ^1^H ions by hydrophobic ^19^F may decrease water solubility, the proposed complexes are highly soluble in water (> 100 mM). The contrast agents are complexes of a tetraphosphinic acid chelator; the hydrophobicity of the –CF_3_ groups is sufficiently offset by the high hydrophilicity of the phosphinates, and, in addition, the complexes have a negative charge.

The structure of the probes is analogous to those of the known tetra-phosphorus acid analogs of DOTA [26], which are similar to the structures of commercially available macrocyclic gadolinium MRI contrast agents [15]. Despite recent findings on Gd toxicity [27] and Gd chelate accumulation in the brain [28], macrocyclic chelates remain as safe compounds for MRI contrast enhancement. They are approved for clinical practice and broadly used for ^1^H MR imaging. In addition, lanthanide(III) complexes of phosphinic acid analogs of DOTA have been proven to be stable in vivo [29]. Therefore, these Ln(III)-DOTP-tfe complexes should have similar in vitro/in vivo stability, tolerance, and renal clearance rate. Although these properties have not been tested yet, a separate in vitro experiment showed that the complex is stable in both phosphate buffer saline and in blood serum for 10 days at 37 °C; no ^19^F NMR signal other than the signal of the complex was observed during this period.

The sensitivity of the investigated complexes was enhanced by combining two commonly used approaches: (a) The molecules contain several equivalent fluorine atoms, 12, and (b) the paramagnetic lanthanide(III) ions shorten relaxation times, thus enabling a higher number of acquisitions in a specific period of time and increasing the signal-to-noise ratio.

The differences in ^19^F relaxation times (moderately decreased by Ce^3+^, Yb^3+^, and very short in Dy^3+^, Ho^3+^ or Tm^3+^complexes) are related to the number of unpaired electrons and to the effective magnetic moments (*μ*_eff_) of the ions as well as to the electronic relaxation. Ce^3+^ and Yb^3+^ have one unpaired electron and low *μ*_eff_ (2.5 and 4.5 BM, respectively), whereas Dy^3+^, Ho^3+^, Tm^3+^ have significantly higher moments (10.6, 10.4, and 7.6 BM, respectively). The effect of lanthanide(III) ions on the ^19^F relaxation of fluorinated complexes has already been described [30, 31], and a model involving chemical shift anisotropy, internuclear dipole–dipole interaction, and Curie relaxation has been presented [32], showing that the distance between lanthanide and fluorine nuclei is a determinant factor.

The relaxation times of the Ce^3+^ and Yb^3+^ complexes made it possible to reduce the TR to 500 or 200 ms, respectively. Moderately long *T*_2_ enabled the use of a turbo spin echo sequence with a high turbo factor. The short relaxations of the Dy^3+^, Tm^3+^, or Ho^3+^ complexes require a gradient echo sequence and make it possible to reduce TR to 15 ms, which enables a substantially higher number of acquisitions (up to 2000 acquisitions during a 16-min sequence) thereby compensating for the signal intensity loss due to the short *T*_2_/*T*_2_* relaxation times. Specifically tailoring the timing to the chelated lanthanide(III) ion made it possible to detect the probes in concentrations as low as 1.25 mM within a 16-min scan. Although the differences in signal-to-noise ratios between the complexes and the empty ligand are not high, the complexes with short relaxation times (Dy, Tm, Ho) may profit from the utilization of ultrashort echo or zero echo time sequences [22], which are, unfortunately, unavailable on our scanner. A gradient echo with TE = 3.19 ms used in our study means a loss of  ~ 90% of the signal due to short *T*_2_*. Ultrashort echo or zero echo sequences would minimize these losses and enable further shortening of the repetition time and increasing the number of acquisitions.

Under in vivo conditions, we succeeded in reliably detecting the Ce and Yb probes (~ 2.7 mM concentration) injected into rat muscle within the same measurement time.

The idea of utilization of fluorinated chelates with bound paramagnetic ions is not new as given in the Introduction. For example, Jiang et al. [30] compared the relaxation times and chemical shift of the complexes with diamagnetic and paramagnetic ions, and proposed their utilization for parallel tracing of several chelates with different chemical shifts. However, due to the long distance between the chelated metal ion and the fluorinated groups, relaxation times of the probes were only modestly shortened.

Based on the chemical similarity between the probes designed in this study and gadolinium chelates broadly used in ^1^H MRI, our probes are predetermined for similar in vivo applications, e.g., as blood pool contrast agents. Although these probes will unlikely compete with the fluorinated nanoparticles [11, 33] as labels for cell tracking various applications [34, 35], in their current form, they may broaden the current range of applications. In addition to the non-specific systemic applications, further functionalization may increase the specific uptake of the probes in various tissues, or their binding to serum proteins may prolong the probe circulation. Furthermore, these probes based on the complexes with different lanthanide(III) ions may also be simultaneously detected and differentiated due to their different chemical shifts [30].

## Conclusion

We designed, synthesized, and successfully tested, both in vitro and in vivo, novel paramagnetic ^19^F MRI contrast agents based on a DOTP derivative with 12 chemically and magnetically equivalent fluorine atoms. The imaging sequences for in vivo applications were optimized with regard to their actual relaxation times. Moreover, these probes were visualized in a rat model, thus proved their applicability to preclinical imaging.
